# Efficacy of platelet-rich plasma impregnation for unidirectional porous β-tricalcium phosphate in lateral lumbar interbody fusion: study protocol for a prospective controlled trial

**DOI:** 10.1186/s13063-022-06857-x

**Published:** 2022-10-27

**Authors:** Kosuke Sato, Toru Funayama, Hiroshi Noguchi, Tomoyuki Asada, Mamoru Kono, Fumihiko Eto, Yosuke Shibao, Kosei Miura, Naoya Kikuchi, Tomokazu Yoshioka, Hiroshi Takahashi, Masao Koda, Masashi Yamazaki

**Affiliations:** Department of Orthopedic Surgery, University of Tsukuba, 2-1-1 Amakubo, Tsukuba City, Ibaraki, Japan

**Keywords:** Platelet-rich plasma, β-tricalcium phosphate artificial bone, Lateral lumbar interbody fusion, Fusion rate

## Abstract

**Background:**

The use of platelet-rich plasma has been increasing in spinal fusion surgery. However, the efficacy of platelet-rich plasma in lateral lumbar interbody fusion is unclear. In Japan, Affinos,® (Kuraray Co., Tokyo, Japan), a β-tricalcium phosphate artificial bone, has been increasingly used for spinal fusion. The purpose of this trial is to demonstrate whether Affinos® impregnated with platelet-rich plasma can achieve a higher fusion rate and better clinical outcomes than Affinos® alone.

**Methods:**

The current study is a prospective randomized controlled trial. This trial will include consecutive patients scheduled for lateral lumbar interbody fusion. An intervertebral cage for lateral lumbar interbody fusion has two spaces for bone grafts. As a consequence, two bone grafts are inserted at each intervertebral level. In this study, an artificial bone with platelet-rich plasma will be inserted into one space, and an artificial bone without platelet-rich plasma will be inserted into the other space. We will compare the fusion rates between the bone grafts with and without platelet-rich plasma. Our primary endpoint will be the interbody fusion rate at 1 year after surgery.

**Discussion:**

This trial will verify the efficacy of platelet-rich plasma with Affinos® for bony fusion in lateral lumbar interbody fusion. It will also provide substantial evidence for the effectiveness and safety of platelet-rich plasma in spinal fusion surgery.

**Trial registration:**

Japan Registry of Clinical Trials (jRCT) jRCTb032200199. First registered on 13 November 2020. jRCT is approved as a member of the Primary Registry Network of WHO ICTRP.

## Background

Spinal fusion is a common surgery performed for spinal degenerative diseases, spinal fractures, and spinal deformities. The use of the lateral lumbar interbody fusion (LLIF) procedure for the treatment of lumbar spinal pathologies is increasing owing to the associated advantages of this procedure [1]. In Japan, artificial bone is used mostly as graft material for LLIF [2]. Although surgical outcomes associated with spinal fusion have improved owing to innovation in spinal instrumentation [3], bony fusion is essential to prevent pseudoarthrosis.

Affinos® (Kuraray Co., Tokyo, Japan) is a β-tricalcium phosphate artificial bone with a porosity of 57% consisting of a novel unidirectional porous structure, in which intercommunicating holes of 25–300 µm are arranged in one direction [2]. In Japan, the use of Affinos® has been increasing for spinal fusion with acceptable results, while the use of an autologous bone as a bone graft is commonly applied in many fields requiring bony fusion [2, 4].

Platelet-rich plasma (PRP) is blood plasma containing concentrated autologous platelets containing several growth factors such as platelet-derived growth factor (PDGF), transforming growth factor-β (TGF-β), and insulin-like growth factor (IGF) [5–7]. Therefore, PRP is used to accelerate bone and soft tissue healing not only in oral dentistry, dermatology, ophthalmology, and sports medicine, but also in spinal fusion surgery [8–11].

The question remains whether PRP can accelerate bony fusion in LLIF. Additionally, to the best of our knowledge, there have been no reports demonstrating the efficacy of PRP combined with β-tricalcium phosphate artificial bone in LLIF.

The purpose of this study is to demonstrate whether Affinos® impregnated with PRP can achieve a higher fusion rate and better clinical outcomes than Affinos® alone.

## Methods

### Aim

To demonstrate whether Affinos® impregnated with PRP can achieve a higher fusion rate and better clinical outcomes than Affinos® alone in the treatment of lumbar spinal pathologies using LLIF.

### Design of the study

The study is a prospective controlled superiority trial study with randomly allocated groups.

### Study procedures

**Fig. 1:**
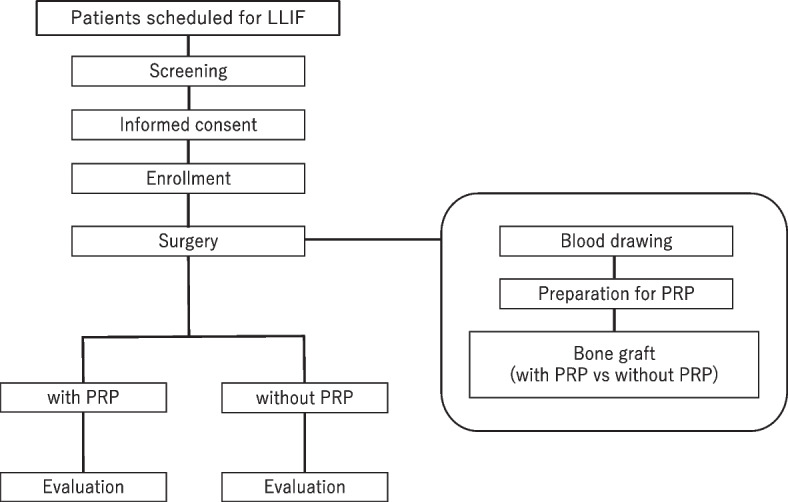
Schematic diagram showing the trial timeline. Consecutive patients scheduled for lateral lumbar interbody fusion will be recruited for this study. Whole blood will be drawn from each patient to prepare platelet-rich plasma. In an intervertebral cage, one space will be filled with a bone graft impregnated with platelet-rich plasma, and the other will be filled with a bone graft without PRP

The outline of this study is shown in Fig. 1. Consecutive patients scheduled for LLIF in the University of Tsukuba hospital will be enrolled in this trial. The target sample size for this study trial is fifteen patients, with a total of 40 intervertebral levels. This trial is approved by the institutional review board of the University of Tsukuba Hospital, and consent for participation in the trial will be obtained from all patients (jRCTb032200199).

GPS® III (Zimmer Biomet, Warsaw, IN, USA) will be used to prepare a PRP. A total of 52 mL of peripheral blood will be drawn from each patient before the surgery. The withdrawn blood will be centrifuged for 15 min at 3200 rpm. After elimination of platelet-poor plasma, PRP will be obtained by extracting the buffy coat layer.

**Fig. 2:**
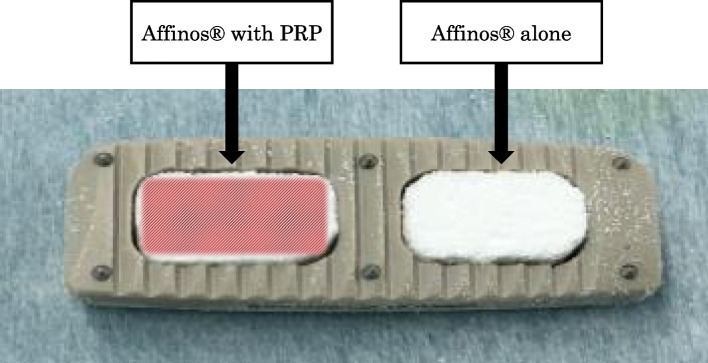
Bone grafts in the intervertebral cage. For each cage, one space will be filled with a bone graft with PRP, and the other will be filled with a bone graft without PRP

CoRoent XL PEEK cage® (NuVasive, San Diego, CA, USA) will be used as an intervertebral fusion cage for the LLIF. A block-type Affinos® will be used as a bone graft in the intervertebral cage. Each cage has two spaces for a bone graft; one space will be filled with an artificial bone block impregnated with PRP, and the other will be filled with an artificial bone block without PRP (Fig. 2). Therefore, two artificial bone blocks can be grafted at each intervertebral level. The side of the bone graft with PRP will be alternately set in the order of insertion. In this way, we will assign interventions (bone graft with PRP) and controls (bone graft without PRP) randomly.

Our primary endpoint is the interbody fusion rate at 1 year after surgery. Plain radiographs and computerized tomography (CT) multi-planar reconstruction coronal and sagittal images will be obtained for the evaluation of interbody fusion at 1 year after the surgery. Trabecular bone continuity between the grafted bone within the window of the cage and vertebral endplates will be evaluated by three spinal surgeons. Interbody fusion will be determined when no instability is identified in flexion–extension radiographs, and bony continuity will be identified in at least one sagittal or coronal slice of CT multiplanar reconstructed images [2].

The secondary endpoints are as follows: (1) the interbody fusion rate at 6 months after surgery; (2) temporal changes in grafted artificial bone such as resorption and remodeling; (3) bony contact between the cage and vertebral endplates; and (4) visual analog scale (VAS), Japanese Orthopedic Association Back Pain Evaluation Questionnaire (JOA-BPEQ), Oswestry disability index (ODI), and European Quality of Life 5 dimensions (EQ-5D).

### Inclusion criteria

The inclusion criteria are as follows: (1) patients who are scheduled for LLIF for a lumbar degenerative disease; (2) patients who are scheduled for LLIF between 1 and 4 intervertebral levels; (3) patients aged > 20 years; and (4) patients who agree to participate in the current trial and from who informed consent has been obtained both orally and in writing.

### Exclusion criteria

Exclusion criteria are as follows: (1) patients with a history of lumbar surgery, infectious spondylitis, and spinal tumor; (2) patients administered another trial drug within 3 months before the commencement of this study; (3) patients with comorbidities, such as abnormal bone metabolism and hemodialysis, that cause inferior osteogenic ability; (4) patients with uncontrolled diabetes mellitus; (5) patients with anemia (hemoglobin < 9.0 g/dL); (6) patients with hematological disorders; (7) patients who are pregnant or nursing; (8) patients with metallic allergies; and (9) patients who cannot provide a written consent.

### Relevant concomitant care and interventions

Intervertebral cages other than those mentioned above will not be used in the trial. No provision regarding the screws and rods for posterior fixation has been set.

### Criteria for discontinuing the trial

Participation of patients in this trial will be discontinued in the following cases: (1) when the patient declines participation in this trial or withdraws consent; (2) when the patient is found to be ineligible for participation; (3) when it is difficult to continue the trial owing to the onset of a new disease; (4) when the patient’s compliance is poor; (5) when the trial itself is discontinued; and (6) when the patient is determined to be ineligible by the doctor for any other reason.

### Missing data and data from participants who discontinue

All patients will be followed up as per the normal postoperative course, ensuring necessary data acquisition for the research. Additionally, to avoid missing data and to promote participant retention, data managers will announce the required tests, questionnaires, and their schedules to attending doctors and patients. Missing data will not be supplemented, and rejected data will be excluded.

### Data management

The personal information of patients, including medical information, will be handled in accordance with the privacy policy of the regenerative medicine provider. Each patient will be issued an ID that will permit tracing of the patient but will also protect their personal identification information using a strictly monitored ID management system.

Data managers will use the case report system to enter data. The case report system will include paper media and an electronic data capturing system. Study coordinators will verify the accuracy of data entry and check for the presence of missing data. An independent data monitoring manager, with no conflict of interest in this study will be responsible for data monitoring at the primary level.

All investigators will minimize the risks of potential adverse events. All related adverse events will be documented, and appropriate treatment will be immediately provided. An interim analysis will not be conducted.

### Confirmation of implementation

The provider, manager, and principal investigator will confirm that the trial is appropriately implemented. They will also confirm that regenerative medicine is provided in accordance with the regenerative medicine provision plan.

### Sample size estimation

The target sample size for this study is 15. Since patients will individually undergo LLIF at 1 to 4 intervertebral levels, more than 40 intervertebral levels are expected to be included in this study. Therefore, at least 80 bone graft spaces will be included to evaluate bony fusion.

A sample size calculation was performed based on the results of previous studies [2, 12]. In previous studies, the fusion rate of LLIF with Affinos® was 70.9%. And, the intervertebral fusion rate with autologous iliac crest bone was 94.5%. Based on these parameters, we calculated a sample size of 40 bone graft spaces per group with a power of 80% and an alpha of 5% (two-tailed).

### Statistical analyses

For baseline characteristics, summary statistics will comprise frequencies and proportions for categorical variables, whilst means and standard deviations will be used for continuous variables. Patient characteristics will be compared using a *χ*^2^ test for categorical variables and a *t*-test or Mann–Whitney *U* test for continuous variables.

The intervertebral fusion rate in each cage space will be calculated using primary endpoint analysis. A *χ*^2^ test will be performed to compare the fusion rates between bone grafts with and without PRP.

For secondary endpoint analysis, a *χ*^2^ test will be performed to compare the fusion rates between bone grafts with and without PRP at 6 months. Multivariate analyses will be performed to assess the relationship of bony fusion with VAS, JOA-BPEQ, ODI, and EQ-5D scores.

Statistical analyses will be performed using SPSS Statistics version 27.0 (International Business Machines Corporation, NY, USA). All *p* values will be two-sided, and *p* < 0.05 will be considered significant.

### Ethics

This trial was approved by the regional ethical review board. The trial will be conducted in accordance with the principles of the World Medical Association (WMA) Declaration of Helsinki.

### Patient informed consent

All patients will be given written explanatory materials and consent forms. The principal investigator will provide patients with sufficient information before obtaining informed consent.

This study will be covered by clinical research insurance. Compensation will be provided to the participants who present with adverse events related to the administration of regenerative medicine in this trial.

### Public disclosure and publication policy

The outline of the trial will be registered on the public registration site, Japan Registry of Clinical Trials (jRCT), prior to the implementation of the trial. jRCT is approved as a member of the Primary Registry Network of WHO ICTRP. The results of the trial will be published in an English journal after the final registration is completed on the jRCT site.

### Protocol amendments

In the case that important protocol modifications are required, approval of the accrediting regenerative medicine committee will be required. The protocol modification, once approved, will immediately be registered on the public registration site, jRCT.

## Discussion

This study is a confirmative trial to elucidate the efficacy of PRP with Affinos® for bony fusion in LLIF. If this study demonstrates the efficacy of PRP, Affinos® impregnated with PRP could be used as an alternative for autologous bone grafts with no risk of complications as opposed to that observed with autologous bone harvest. The current study is important to develop a viable procedure for substantial and safe bony fusion in LLIF.

## Trial status

This trial is currently being conducted. Recruitment of study patients commenced on February 24, 2021. Trial completion will be expected at 1 year after surgery.

## Protocol version

Version. 4.0 (April 15, 2022).

## Data Availability

Only the principal investigator and designated investigators will have access to the final dataset.

## Abbreviations

PRP: Platelet-rich plasma
LLIF: Lateral lumbar interbody fusion
CT: Computerized tomography
PDGF: Platelet-derived growth factor
TGF-β: Transforming growth factor-β
IGF: Insulin-like growth factor
VAS: Visual analog scale
JOA-BPEQ: Japanese Orthopedic Association Back Pain Evaluation Questionnaire
ODI: Oswestry disability index
EQ-5D: European quality of life 5 dimensions
jRCT: Japan Registry of Clinical Trials
